# Physiology and Hygiene of Persons Who Devote Themselves to Intellectual Toil, &c.

**Published:** 1841-04-01

**Authors:** 


### PHYSIOLOGIE ET HYGIENE DES HOMMES LIVRÉS AUX TRAVAUX DE L'ESPRIT, &c. Par *J. H. Reveillé-Parisæ*, Docteur en Medicine, &c. Paris, 1840. PHYSIOLOGY AND HYGIENE OF PERSONS WHO DEVOTE THEMSELVES TO INTELLECTUAL TOIL, &c.

IT is generally believed by those who support life by corporeal labour, and eat their bread in the sweat of their brow, that physical inactivity is downright idleness. Such persons can have no conception that weariness can be contracted in an elbow-chair, by now and then peeping into a book, and musing the rest of the day. Hence it is that the sedentary and the studious raise their envy or contempt, according as they appear to possess the conveniences of life, by the mere bounty of fortune, or to suffer the want of them by refusing to work.

It is, however, certain that to think is to labour; and that as the body is affected by the exercise of the mind, the fatigue of the study is not less than that of the field or manufactory. But it is equally certain that the labour of the mind is not attended with the same advantages. Exercise gives health, vigour and cheerfulness, sound sleep, and a keen appetite; while the effects of sedentary thoughtfulness are diseases that embitter and shorten life; interrupted rest, tasteless meals, perpetual languor, and causeless anxiety.

In gratitude to men of literary pursuits for the sweet enjoyments they afford us by their intellectual toils, we are in duty bound, as medical men, to tender our advice to them, and to point out to them, the dangers to health and to comfort, and to every thing that can make life valuable, inseparable from excessive exertion of the brain—to show them, in fact, how much the noble functions of intellectual life wear out and debilitate material life; and how destructive the labour of thought is to physical existence. Nature, anxious for the preservation of this existence, has traced out laws, the rigorous observance of which is to be found only in the infancy of society, or in the habits of savage life: the empire of these laws decreases as civilization progresses; and when, by this progression, the study of the sciences has become generally diffused, we see their adventurous and enthusiastic admirers pursue them at the expense even of life itself. Now in virtue of the order established in the creation of living bodies all the organs mutually aid and balance each other; nature has attached enjoyment and health to the regular and free exercise of all the functions, whilst she visits with the tortures of disease the too frequent or too prolonged exercise of any one organ, and the predominance of any one physical or moral function.

Every man who observes and reflects will, nay must, admit that a reciprocal action takes place between our physical and moral condition. Of such sympathies, like many other mysteries of nature, *causa latet*, the cause remains concealed, while the effects are palpable and obvious. This close, yet inscrutable association, this latent correspondence of parts seemingly unconnected, this reciprocal influence of mind and body has long fixed the attention of medical and metaphysical enquirers. Can we, says a modern writer,* conceive the mysterious inhabitant as forming a part of its own habitation? the tenant and the house are inseparable, so that in striking at any part of the building you inevitably reach the dweller.

If we look around and survey animated nature, we shall find that man, and more especially civilised man, is of all animals the one most liable to disease. How then must it be with those men who carry within themselves the very moving, impelling, and creative principle of civilization? Every thing, which bears on social man, re-acts on their physical, as well as on

---

* D'Israeli's Curiosities of Literature.

their moral constitution, with an intensity almost always prejudicial to their well-being; every thing here combines to become an immediate cause of disease. A delicate organisation; or one rendered so by intellectual toil; extreme sensibility; and an incessant and habitual exaltation of this same sensibility; an imagination ever in a state of tension; the energies of the brain continually in action; a neglect and forgetfulness of those cares and attentions necessary to the preservation of health; what a crowd of causes calculated to destroy the springs of the human economy, and to undermine its strength; to render the body languid and consequently obnoxious to the effectual attacks of morbific agents; to convert life, in fact, into a continuous fever and a never ceasing struggle!! All diseases, to which the human race is liable, may become developed in men devoted to extreme intellectual labour. The elements forming their constitution, nay, their very thoughts, contain within them the germ and principle of a multitude of diseases, irritability, the great characteristic of deep thinkers, being the grand element which predisposes to inflammatory and nervous disorders. However, as every temperament has a particular tendency to some particular order of diseases, it is also observed in the case of studious and deep-thinking men, that some pathological affections are more frequent in them than others. Thus the delicate frame of Kirke White, who gave such extraordinary promise of great poetical genius, was doomed to become an early victim to pulmonary phthisis, the progress of which, though the seeds were probably laid at an early period of existence, was no doubt accelerated by the ardour and enthusiasm with which he prosecuted his favourite studies.

> " 'Twas his own genius gave the final blow,
> And help'd to plant the wound that laid him low."

We fancy we hear some laborious and enthusiastic student, whose declining health, induced by incessant study, has already awakened the alarms of his anxious friends, reply to their fond remonstrances, when dissuading him from his destructive course, in the indignant language of the Roman Satirist—

> Et propter vitam vivendi perdere causas!!

Yes: with such an individual, mere physical life is not worth having; it is only in the intellectual and metaphysical regions of abstract meditation and intense thought he feels that he

> ——— " lives, and moves, and has his being."

The mere matter-of-fact person, however, who attaches some importance to the condition in which the mind's tenement is kept, who condescends to feel that there is some sympathy between his mind and the body, and that on this sympathy much of the happiness and misery of ordinary mortals mainly depends, such person, entertaining a less sublimated, we admit, but in our opinion, a more correct view of the matter, may reply to the above quotation in the forcible language of Martial—

> Non vivere, sed valere, vita est, ————

Having thus introduced the subject of our author's work to the notice of our readers, we shall proceed to give an analysis of its contents.

The object of the author, in the first part of his work, seems to be to reduce every thing, no matter how refined in intellectuality, to the property or function of sensibility, and thus to explain by the laws of this function the moral existence of men the most distinguished for their works and their genius. These men, he says, predisposed to lively sensations and ardent emotions, (for in them the impression surpasses in intensity and duration that which occurs in ordinary mortals,) become voracious of these very sensations. By the mass of ideas which they acquire in a short time, they soon attain a vast store of knowledge; then endowed with a capability of expressing this, and carried away by their own thoughts, they feel an irresistible desire of communicating them to the external world. These thoughts it is which impose laws on kingdoms, become the vivifying strength of those mighty souls destined to civilise, elevate and degrade nations. Cromwell, in his time, was the visible destiny of the moment, as Napoleon was in ours. Now, he asks, how is such vital and intellectual activity compatible with tranquillity and regularity of the system? Is not life in excess here, as well in the moral as in the physical being? Observe, accordingly, that incessant inquietude, that unremitting and restless activity, that internal ebullition, which is every instant disturbing the organic powers, that feeling of life so intense and occasionally so painful, which imparts to the existence of distinguished men something violent, restless, feverish and inexplicable, something, in fact, altogether out of the routine of ordinary life. This state of inquietude either ceases altogether, or at least diminishes, when life is very active, or when the torrent of ideas is allowed to flow. Such a crisis is in general salutary. It is then those master-pieces are produced, then it is those treasures of the imagination are poured forth for the relief and satisfaction of the individual's self. Poetry is in the poet just as sound is in the lyre; this is a positive philosophical truth.

Men of genius often work without caring what becomes of their work, and merely to satisfy themselves. Our author next anticipates an objection which may be urged against these principles; namely, that they hold good only with respect to artists, whose imagination is more ardent than that of the savant. This, however, he conceives to be a mistake. The savant, he says, who is endowed merely with the capability of knowing, is but a mere man of learning; he knows what has been already done; but when possessed of high intellectual faculties, should he wish to extend the boundaries of science, he investigates, invents, and *imagines.* In this view he is fully borne out by every day's observation. There is no individual in the social, civil, or philosophical world, who has more frequent occasion to draw on the stores of imagination than the natural philosopher, in order that he may be able to account for the phenomena presenting themselves in the physical world. Should the facts refuse the imagined explanation, it is a mere idle theory or hypothesis: if, on the other hand, the facts correspond with it, and the theory so imagined be but the expression of them, a progress is made. To seize on a general principle, to investigate its most remote consequences, and to trace them with a force and vigour of thought capable of attaining great results; then to express and generalize this principle, so as to render it applicable to every thing which may be deduced from it; such an intellectual performance is but the flight of a powerful imagination. Homer and Archimedes were in the same category with respect to invention. So that this ardent sensibility of soul, which impassions all its ideas, is found as well in the philosopher as in the artist; it is the same enthusiasm for their works, their conceptions, their theories, or their systems. In fact there is just as much of vivid imagination in first conceiving such hypotheses as the Anima of Stahl, or the Archæus of Van Helmont, as in imagining the most unsubstantial creations of the most fanciful poets; with this difference, however, that stubborn facts may sometimes be adduced to test the truth of the physiologist's imaginings: from which ordeal the poet is quite secure.

Our author now goes on to shew that, in consequence of this intense feeling, these existing and powerful emotions of great minds, the faculties sometimes become debilitated. The soul, as it were, becomes dried up, just as the body of the sensualist is enfeebled and worn out. The cause of these phenomena is one and the same. However superior the organization of the nervous system may be, it cannot go beyond certain limits. The intellectual and moral life, though the primary, essential and true life of man, yet like every vital act, requires to be kept within certain bounds. Should we wish to give to the faculties of feeling and of knowing an unlimited extension, the organism soon refuses to respond to such exertion; it becomes enervated more or less rapidly. Then it is the man of genius becomes a prey to his own extravagant ideas. He still desires; but what does he desire? what does he wish? why does he sigh? he knows not. This ardent aspiration of the faculties after something undefinable and increate, such a soul, sometimes carried up to the third heaven, sometimes saddened and depressed even unto death; those flights of a delirious, restless imagination, without apparent end or determinate object, have been frequently observed and as frequently described. Such a state really exists in certain individuals endowed with great moral energy exercised too early and without moderation.

An additional proof that this extraordinary state depends on sensibility prematurely exhausted is, that the imagination, no longer finding any external element, turns in upon itself, and makes prodigious efforts to combat the evil effects of ennui and of too much thinking. Flying from one abstraction to another it ultimately rests confiding in the truth of Rousseau's oft repeated maxim:—Il n'y a rien de beau que ce qui n' est pas. Now the first origin of this is an extremely excitable nervous system, an incessant and active sensibiliy. The most out-and-out spiritualists are often reduced to this same consequence in spite of themselves. Pascal says truly, "we must not forget ourselves; we are body as well as mind." Plato, that prince of spiritualists, so famed for conceiving and embodying the most sublimated abstractions, says, that "every pain, every pleasure, has, as it were, a nail, whereby it fastens the soul to the body, assimilates it to itself, and makes it believe that nothing is true but what the body has told it."

Nature has then wisely ordained that the harmonic play of our sensations should be successively excited with various shades of activity and strength; but she at the same time apprizes us, that it is madness to desire superhuman impressions with our present state of organic debility, or to require of life more than life can give.

### THE TWO WAYS IN WHICH LIFE MANIFESTS ITSELF.

Without infringing on the principle of unity, it may be said that life presents itself under two general and perfectly distinct modes, *sensibility* and *contractility*; the former depending on the nervous system, the latter on the muscular system in general; these two modes of vital manifestation have been called *innervation* and *locomotion*, the *sensitive powers* and the *motive powers*. These two properties are found in all the phenomena of life, though in different degrees; their development is often in an inverse ratio, yet it is sometimes simultaneous. After pointing out the progressive perfection of the nervous system from the individuals constituting the lowest class of animality up to man, in whom it becomes the type of the most perfect organisation, and after shewing the vast importance and immense extent of the diversified ramifications of this system, whereby it surrounds the entire economy in a sort of nervous atmosphere, our author comes to the enquiry as to how impressions take place, and how the cerebral influence is propagated—he here enumerates the various hypotheses which have been given in order to account for this effect; but acknowledging the unsatisfactory state of our knowledge on this point, he gives up the investigation, and next comes to the consideration of SOME OF THE MOST GENERAL LAWS OF SENSIBILITY.

The *action* of the nerves has for its results sensibility; that is, an aptitude to receive impressions, whether from the external world, or from the organism itself. These impressions, transmitted to the individual, *to self*, become perceptions, intellectual and moral acts; and these acts, in their turn, manifest themselves externally by a re-action of the nervous centre on the periphery. Thus, on the one hand, we have impression, transmission, action; that is, an intelligence which knows, a will which determines, and a power which acts: such is the fundamental law of sensibility, considered in its greatest possible extent. And thus our author will have it, that this property of living beings is not a mere passive property, a mere *receptivity*, according to the doctrine of Kant and his followers, but that there exists a proper and palpable activity of the nervous system taken as a whole. This system, then, being the material and indispensable condition of the modifications of thought and feeling, the measure of its perfection will be the measure of the intellectual faculties in the scale of beings.

It is, our author observes, to this system that man owes his superiority. The study of the nervous system considered from one animal to another, presents immense differences, both in an organic and mental point of view. Nor is it in the different classes of animals only these differences are remarked, but also in individuals of the human species. Thence results an extreme variety in the capacity of feeling, and consequently a greater or less development of the intellectual faculties. Generally speaking, we appreciate the energy of life by the force, duration, and frequency of our sensations. Now the more adapted the organ is to feel, the more marked in us is the desire of being moved, and of being, as it were, apprised of our existence; there are some persons who cannot be satisfied in this respect; they indulge in this desire even at the expense of their well-being and health. On what, our author asks, does this *voracious appetite* for sensations and mental emotions depend? on nothing else than a very complicated nervous apparatus, endowed with an extraordinary capacity tor feeling; a capacity which is only increased by the multiplicity of the impressions made on it. This desire of mental emotions is more especially remarked to exist among civilised nations, and is justly considered to constitute the elementary principle of the fine arts. Harmonious poetry, enchanting music, are judged to be such only according to the sensations which they excite in the reader or hearer. It is the very perfection of art when the reader is so swayed, so completely subdued, as that he no longer knows whether he holds a book in his hands. In what does this great, this grand secret consist?—in multiplying the impressions, in striking the imagination, in, as it were, pricking the nerves with the stings of the mind. Thus the sphere of action of the nervous system, as of all the other systems, increases with the extent and perfection of this same system. There is another law influencing the sensibility of relation, which is not of less importance; it is this, that it manifests itself in two ways, *pleasure* and *pain.* The former includes every sensation which we would wish to prolong, and which seems useful to the system; whilst the latter includes every sensation which we would wish to repel, as injurious to existence. And is it not obvious how well the organic movements accord with this explanation? Should the sensation be pleasing, instantly all the movements become expanded, the tissues unfold themselves, nature seems to present the greatest possible extent of surface, so that no part may escape the impression; whilst in pain, on the contrary, these same movements become constrained, the tissues contract, nature presents a *minimum* of surface to the enemy, as if she wished to escape him, or as if she wished to concentrate her strength to combat him. Pleasure and pain are real elementary sensations, the two poles of sensibility; for the other sensations are but shades of them. It may be remarked, also, that what is called *moral sensibility,* in a similar manner presents only two primary or fundamental feelings, namely, love and hate; both being the grand principle of our passions, whether such passions be of an exciting or depressing kind. Accordingly pain and pleasure have but one common origin, and are therefore closely connected. There are painful sensations which are not without their charms; and at the extreme of pleasure, pain commences. The latter, as we see, is necessary and indispensable to the regularity of the functions, it being, as it were the exciter of the conservative principle, the signal and cry of the suffering organ; nor has it, indeed, been clearly shewn which is the more injurious or the more useful to man, pleasure with its roses, or pain with its thorns.

Whatever be the activity of these two sensations, neither of them can be continued, intermission of action being one of the characters of sensibility. Like to all the other functions, cerebral sensibility, with which we are at present more immediately concerned, presents alternations of rest and of action: these intermissions are necessary for its reparation, which is completed only by sleep. This law of intermission of action now under consideration is of the highest moment not only for bodily health, but also for mental or intellectual energy, and for every thing connected with cerebral action.

It may again be observed, that this intermission not being complete, nervous action never presents that character of uniformity and steadiness remarked in other functions. Mobile, inconstant, exceedingly variable in its intensity and energy, sensibility often passes, with astonishing rapidity, from the lowest degree of prostration to the highest point of exaltation. It is a free, an *independent* faculty, as incalculable in its effects, as unknown in its cause. Alternately ardent, strong, prostrated, exalted, it pervades and stimulates certain organs, and suddenly abandons them for others. Its proportions never continue the same in any one organ; fixity, permanence, or precision of action are never to be predicted of it.

This law of *mobility*, which is so important, gives rise to another no less essential to be known, we mean the law of *concentration*. It has been said that it was with sensibility as with a fluid of a given, determinate quantity; which, if it flow plenteously in one of its channels, becomes proportionally diminished in the others. The comparison is by no means deficient in justness. It is certain that the more an organ is excited, the more sensibility becomes accumulated in it, and always at the expense of the sensibility in other organs. This simple law of physiology, observed from remote antiquity, is probably one of the most fruitful with respect to disease, hygiene and philosophy. Confining our remarks to the intellectual faculties, we may observe, that this law of concentration is what is usually called contemplation or meditation, which is combining or bringing together all the data on any subject whatever, so as to examine them, and to discover all their relations, and to deduce consequences from them. These consequences are then applicable to the arts and sciences. Thus thought becomes the greatest of human powers. If genius be, as it were, the focus of a burning glass, which throws heat and light only on one point, it must certainly be so by its power of condensing the nervous action with the greatest possible force. Such is partly the origin of the high intellectual faculties, the happy or sad prerogative of certain men destined to agitate, excite, and transfix inferior minds. It may be objected, that we are here confounding the action of the brain with sensibility in general. To this we reply that the nervous system is identical in all its parts, and that as sensitive unity or unity of sensation is indispensable to unity of being, since it is what constitutes it, there must be some one organ to sympathise with all, to communicate with all parts of the system; this organ is the brain. Nor is it less true, however, that if this organ is always the point of concentration of sensibility, the other organs soon become changed by the deficient supply of that innervation which is necessary to their normal action. This is one of the causes of the loss of health in those who exercise the mind immoderately. We already see the origin of those evils which torment the man of genius, and which are engrafted as fruits of death on the tree of life.

We may further observe that, in certain cases where cerebral excitement is carried to the highest degree, the other organs are almost insensible to external impressions. The soul no longer perceives any thing external; and the individual or *self* seems to be brought back to his state of metaphysical simplicity. Mental abstraction, enthusiasm, contemplative ecstasies, certain diseases, as delirium, or catalepsy, shew that these phenomena are not uncommon. They are also observed in those engaged in profound meditation. Archimedes, when intent on solving a geometrical problem, was, one might say, a mere intelligent abstraction, when the Roman soldier struck him. Tertullian well said of the enthusiastic martyrs of the Christian faith: *nihil crus sentit in nervo, cum animus in cœlo est.* Such a state however cannot last: the disproportion of the innervation is too great not to destroy the equilibrium of the functions.

What has been now said of the variations and oscillations of sensibility in general, is equally observable in the intellectual faculties, more especially when they are very active and very much developed. There is one in particular, which presents this character in the most striking manner, and that is the imagination. There is no intellectual faculty which presents more variety in its energy, in its degrees of depression or elevation than this. What some have called its *prism* is nothing but the different modifications of an extreme cerebral sensibility. The mobility, and inconstancy of the imagination are the facettes of this prism, the reflexions from which convey to the soul alternately and with surprising rapidity, the sensations of joy the most lively or of melancholy the most profound. Moral sensibility, like its great origin, physical sensibility, is subject to certain laws. Rigorousness of method, fixity of rule, are never applicable to it, especially when it is active and predominant. If it be asked whether calmness of the senses, tranquillity of the heart, the gentle undulations of thought, which prove so well the moral and intellectual harmony, are attributes of beings eminently sensitive: we say, undoubtedly not. And the reason is, because the force of impulse, the exciting power, is always either too weak or too energetic, the difference of the effects being directly proportioned to the variability of the principle of action. This explains why the character of individuals strikes one at once by certain inequalities invariably owing to the numberless fluctuations of sensibility. It has been truly said that a long catalogue might be drawn up of the terrors felt by the brave, and of the absurdities fallen into by the man of mind; that it is physically impossible to continue a great man from morning till night.

Very remarkable effects oftentimes result from the excitations and these extreme and continual variations of sensibility. The one is a complete exhaustion of this property, a total prostration of the physical and moral powers; whilst the other, and one which is much more common, is that the nerves acquire so great an excitability, that the slightest stimulus will occasion a nervous action totally disproportioned to its cause. The consequence of this is, that the *intensity* of the sensation will depend less on the intensity of the cause, than on the disposition of the individual.

This state of extreme irritability, when carried to its highest pitch, is justly considered a disease. It has been further observed, that acute senses, excessive nervous irritability, feelingly alive to the slightest physical and moral impressions, will generally, if not always, be found to correspond with a mobile, irritable, and inconsistent character. Such a constitution again re-acts on the organs and disturbs their functions; the mind every instant throws its incasement into confusion. Every one must have observed such a person, either suffering or fancying that they suffer, without being able to assign the slightest cause for it.

Sensibility is thus the property *par excellence* of organized living beings; it attains its highest amount of activity in man; it is by it he exists, acts and lives; in a word, sensibility is, as it were, the material of which life is made.

This property, however, is not merely the moving principle of organic action; through the medium of our consciousness it becomes the source of our pleasures and pains; it influences the character, the propensities, the will, the ardour or weakness of the imagination, the violence or moderation of our desires, the activity or inertness of the intellect. Considering the matter physiologically, we may say that man is what sensibility has made him.

The second form in which life manifests itself is contractility; a property which resides more especially in the muscular system, though always under the influence of innervation. The most contractile organ in the body is also the most sensible. The heart is of all muscular organs the most irritable, and it is also that which seems most under the immediate influence of the brain—hence it may be fairly inferred that sensibility and contractility have their origin in the nervous system. We know that a violent electric shock, or poisoning by prussic acid can destroy both, and that in this way life is arrested at its very source.

We may remark, however, certain differences between these two properties with respect to their mode of action, and the phenomena which they present.*

Sensibility receives impressions and transmits them; contractility triumphs over all external obstacles, whether instinctively or consciously. Sensibility, a careful sentinel, keeps watch within and without; but it is contractility alone which acts and re-acts. Properly speaking, sensibility or nervous power is the regulating principle, which warns, directs and commands, whilst contractility, or muscular power, is the agent which obeys and executes; it is, as one might say, strength personified. These two properties are mutually dependent on each other. Without contractility, sensibility would be devoid of result, action, or influence; without sensibility, contractility would have no mover, nor director. Sensibility is the first called into play; it appreciates the relation which exists between the exciting body and the degree of organic re-action requisite, whilst it is to contractility that such re-action is confided. The animal and vital functions come within the domain of contractility. We may here observe that sensibility, when exalted to a high degree, is capable of communicating to contractility an extraordinary, though always irregular, amount of energy, as we sometimes see in the cases of maniacal and hysterical patients when they are convulsed.

### THE FUNDAMENTAL LAW INFLUENCING THE TEMPERAMENT OF PERSONS DEVOTED TO MENTAL EXERTION.

If all parts of the human body possessed the same degree of energy; if we could attain a uniform and constant equilibrium of the organic powers, there would then be no such thing as *temperament*. Then we should have the *beau ideal* of physiological symmetry; but such a thing does not exist. One or more systems of the animal economy, or some one important organ,

---

* Of the anatomical difference of the two orders of nerves on which these two properties depend, we shall take no notice, for obvious reasons.

obtains the mastery, and this gives rise to what is called *temperament.* When to this physiological condition other circumstances are added, such as peculiar habits of life, education, climate, or diseases, such temperament becomes modified; it becomes increased or diminished according to the nature and direction of the causes now mentioned.

Of all the powers of the system those probably which present most variety in their energy, are the *sensitive* and *motive* powers. Originally established for the purpose of aiding, balancing and moderating each other, in order to the preservation of health, it seldom happens that such action is duly proportioned. To feel and to act are the prominent duties or business of life; our preservation in *being* and well-being depends on the prudent combination of their action. But how difficult it is to keep them within the limits compatible with health! In some persons the sensitive powers obtain the mastery; they acquire, sooner or later, a marked predominance: the nervous apparatus is then endowed primarily and originally with a great capacity of action, a capacity, which only increases by the very inordinate exercise of this power. This increase is in conformity with a physiological law according to which an organ continually in action progressively acquires additional strength, energy and preponderance. But, on the other hand, and in virtue of the same law, if the sensibility is more active, contractility diminishes in the same proportions. The consequence of this is, that the nervous system attains the ascendency in the animal economy, the vital powers become concentrated towards it, whilst the contractile vigor of the organs ceases to be proportioned to this abnormal state. Certain functions acquire extraordinary activity, whilst others become languid from defect of innervation. Hence it is the energy of the radical powers of the economy ceases, their distribution being no longer equal. Such phenomena are observable in extremely nervous persons, but more especially in persons who exercise their mental faculties too much. From these considerations the following *law* is laid down:

On the one hand: ORIGINAL NERVOUS DISPOSITION;

Then, EXCESS OF ACTION;

Finally, EXTREME, CONTINUED PREDOMINANCE OF THE NERVOUS SYSTEM:

On the other hand: GRADUAL AND ALMOST ABSOLUTE DIMINUTION OF CONTRACTILITY.

Such, according to our author, is the fundamental law, the organico-vital condition; the predominant and distinctive character of this temperament, to the development and application of which law he destines his work.

This he calls the fundamental law, admitting at the same time infinite shades of it; as it would be wrong to suppose that men devoted to intellectual occupations do not participate more or less in those varieties of constitution described by physiologists. Predominance of the nervous system may, and in fact does, connect itself with all the known forms of temperament, though there be characters peculiar to each of these forms. Thus when this predominance occurs in bilious and melancholic temperament, it presents effects quite different from those occurring in the lymphatic temperament.

Our author here remarks that, notwithstanding the contrary opinion of several physiologists, both ancient and modern, the nervous apparatus may possess great activity, at the same time that the muscular system may have acquired very marked development. It is well known that that prince of philosophers Plato* was famous for his square shoulders, and a vigorous constitution. Similar instances of active sensibility and muscular vigour combined, may be found among the moderns, for instance, Leonardo de Vinci, Buffon, Gluck and Mirabeau. It must be admitted, however, that this happy coincidence very rarely occurs.

This want of correspondence between the motive and the sensitive powers is more especially remarked in certain vital actions. Contractility, instead of moderating and balancing nervous action, becomes on the contrary subordinate to it. This sensibility in excess seizes on the muscles of animal life; it sometimes increases their action in an extraordinary manner when it becomes exalted; thus we see anger doubles and trebles the ordinary strength of individuals; at other times it stuns and paralyses these same muscles. Another effect of the same cause is, that the muscular fibre becoming more and more debilitated, is at length ready to contract on the slightest nervous excitement. Hence that extraordinary tendency to spasms, convulsions, and irregular contraction of muscles, whether of the voluntary class or not, so often observed in nervous and irritable individuals. The movements of such persons are in general impetuous, their gestures quick and abrupt; but it is in the muscles of the face the effects of these involuntary contráctions are principally to be seen, especially when they are excited. Several distinguished men have exhibited these spasmodic symptoms. The Emperor Peter I. was subject to a kind of tic, which however did not frequently recur, but when it did, it affected his eyes, and his entire countenance, so as to render it terrific. Napoleon was subject to an *involuntary* movement of the right shoulder, and at the same time another movement of the mouth from left to right, when under great excitement. In such a constitution contractility is no longer confined to its natural limits, it is debilitated and incapable of acting according to the normal impressions of sensibility; its action being almost always irregular, whilst sensibility, augmented and exalted, predominates over all the functions of the system.

*On the Effects of this Law on the Physique.*—The first and most striking consequence of such an organic conformation is, that, he who has received it from nature, experiences a more keen feeling of existence than other men. He sees much, for he feels much. Great *affectibility*, which is the distinctive sign of this organization, is observed chiefly among poets and artists. Every thing strikes, every thing excites them, every thing is depicted to them with force and feeling. This characteristic facility of emotion and exaltation soon imparts to the whole system a mobility and acceleration in the vital acts, which cause the slightest impression to affect instantaneously the entire economy. There exists, in fact, a focus of life and action, the intense irradiations of which extend to all points of the organism. Salvator Rosa says that a painter is *all spirit, all bile, and all fire*, nor is this language as metaphorical as one might suppose.

It would be wrong to suppose that this vital energy was restricted to the brain itself exclusively. The nervous system is *one*, and consequently the

---

* He was called Plato from πλατὺς, broad, in consequence of the great *breadth* of his shoulders.

phenomena connected with it, are referrible to the parts which compose it, according to the order of their functions. Quick, and clear perceptions, rapidity of conception, require the perfection of all the nervous branches and fibrils. Exquisite delicacy of sensation demands, *à priori*, exquisite sensibility of the nerves. A rich imagination, often depends on a happy and tenacious memory, which itself requires in the peripheric nervous organs an extreme readiness to be affected. The delicate and exquisite feeling of the poet and the artist resides in the nerves, as well as in the brain. So that it is not the latter organ alone which predominates in the temperament now under consideration, but the entire sensitive system. If the visceral nervous apparatus re-acts, the brain is strongly excited; if the re-action commences from the brain, the sensitive powers at once become electrified.

It is now easy to see why this temperament is so mobile, and so readily affected; one might call it a sonorous instrument, which vibrates on the least touch, and by the slightest excitement. It has been well compared to the *Eolian harp*, which sounds on the slightest breath. The privileged persons to whose lot such a temperament has fallen, feel more joy, more annoyance, more love, more dislike, more transport, more ardour, more passions, more good and evil, and more enthusiasm, than beings endowed with an inferior organization. In the chances of human destiny a lot has been assigned to them replete with enjoyments and pains; this explains why all that life contains of pleasures and of cares, of sweetness and of bitterness, seems reserved for them; how it is that they are at one and the same time the weak and the strong among men, the favourites of Heaven, the idols of their own age and of posterity, and yet, too often, the unfortunates of this world. The fact is, it is because they are more men than other men, either for good or evil. If the degree of perfection of the nervous system indicates the degree of perfection in the animal scale, it is certain that there exists in certain beings eminently endowed with sensibility something above the rest of mortals. The physiological pre-eminence is the principle of the intellectual, and consequently of the social pre-eminence. It has been said that great men are the aristocracy of their species, it is true; but the fact has only been asserted; the object of our author is to establish the validity of the claim.

This superiority, however, is well counterbalanced. Why is it, asks our author, that Nature has impressed the seal of humanity, viz. imperfection, on this her master-piece? Two causes, he says, counterbalance these immense advantages. The first is, that the excessive vitality now under consideration, having its source in nervous power, is, like it, variable, irregular, and transitory. Such a state, far from sustaining, consumes and destroys life. There is energy, no doubt, and organic tension; but it is by convulsive starts it shows itself. Such individuals deluded by a certain factitious vigour, fancy themselves strong because they are excited; they know not that there is merely an unequal distribution of nervous power, and sometimes a spasmodic rigidity of the muscles. And as a proof that such is the case, do we not see that such a state lasts but a short time, that it is immediately followed by a *collapse* exactly proportioned to the degree of previous excitement. The delicate structure of men pre-eminently endowed with sensibility could not support this organic super-excitement if continued for any time. Every thing in it seems to evince an antipathy to mere flesh and matter. The man most poetically organised, is in truth devoid of material strength.

The second cause of the deterioration of the *nervous* constitution is the more or less rapid diminution of contractility. We know that it is the nice adjustment of the different movements, and the exact balancing of the sensitive and motive powers that constitute the stability of vital energy. But no such thing is to be found in the constitution now before us. We do not mean to say that the excitement is, generally speaking, always uniform in the animal system; we know it varies more or less in different organs; but in a healthy and well-constituted body the equilibrium in the organic actions is soon restored according to a generally observed law. If however the nervous apparatus have acquired an extreme preponderance, this law no longer holds; the motive powers are degraded and debilitated, when the sensations are too vivid, too rapid, and too diversified and numerous.

Weakness or nullity of the contractile action of the tissues is felt in all the functions of the economy; the muscular system more especially falls into a progressive state of debility, which is always proportioned to the increase of action in the nervous apparatus, more especially if the individual devote himself to a sedentary life. The muscles become pale and diminished in size; the cohesion of their fibres is no longer the same; they often become atrophied; hence organic re-action becomes totally impossible. Nor is it to be imagined that the external muscles of animal life only are thus debilitated, those of internal and organic life share in this debilitating influence; a circumstance attended with the most disastrous consequences to the several important functions.

Every one must have observed, and sometimes with envy, the easy and rapid digestion of robust men, especially when they feel little, and think still less. It is evident here that not only nature is not drawn off by the brain from the great process of alimentary elaboration, but that the accomplishment of this act is accelerated by the powerful contractions of the stomach, intestines, diaphragm and abdominal muscles. Another advantage of this muscularity is that of retaining the food in the stomach for a sufficient time in order that it may there undergo adequate elaboration. The fibrillary oscillations of the muscular planes of the digestive apparatus, that which constitutes the intestinal peristaltic motion, more especially contribute to render the digestion complete; but these muscular strata are always pale, and attenuated, and sometimes scarcely exist at all in very nervous persons. The diaphragm, that powerful and active muscle, so necessary to the internal organs, also loses its contractile property, nor does any thing contribute more powerfully to produce that languor of the viscera observable in sedentary persons. This explains the connexions of several phenomena. On the one hand, we have painful and laborious digestion, hence badly elaborated chyle, impoverished blood, imperfect nutrition, the losses of the system not repaired, and great prostration: on the other hand, the appetite is either altogether gone or becomes fanciful, the sensibility of the stomach is irregular or depraved, and a permanent state of irritability or atony in the digestive passages. Besides it is observed that the biliary secretion is altered in its quantity and in the quality of its products. The liver, sometimes gorged with black and stagnant blood, often participates in this morbid state; it becomes sensitive and painful; it re-acts on the stomach, and the concurrent affections of both are the ground-work of many pathological phenomena whose influence is soon felt on the morale.

Constipation, that daily torment of sedentary persons, and more especially of literary men, and such like, is not a mere echauffement, as is commonly said; when of long standing, it is evidently to be attributed to *muscular atony* of the intestinal canal. *Defecation,* a function so important to health, is effected only by the simultaneous action of the large intestine, the diaphragm, and the abdominal muscles. Is it not evident then, that muscular power alone is called into action in the exercise of this function? *Flatulence,* moreover, which is so annoying a symptom of bad digestion, is decidedly owing to a defect of vigorous contractility in the digestive system. The abdomen becomes tense and swollen, the debilitated organs contained in it being inordinately distended by the intestinal gases.

This diminution of contractility, and the aberrations of the nervous power, likewise influence the circulation. Witness the palpitations of the heart, and the irregular movements of this organ so frequent in irritable and nervous persons. The circulation is sometimes slow, sometimes rapid, occasionally suddenly interrupted, but always unequal, and rarely presents in this temperament a calm and regular rhythm. Sensibility, and consequently the sensations and various emotions, have too powerful, too direct an influence on the heart for it to be otherwise. The imagination, always active, and rarely confined within the narrow limits of the necessary, the real, and the possible, is every moment disturbing the system. It is said that Madame de Staël, in her youth, could not look at a person of distinction without feeling violent beatings of the heart; at so early a period was her state of health changed.

This deficiency of contractile vigour in the circulatory apparatus, accounts for a phenomenon, which always astonishes even medical men; namely, the slowness and weakness of the pulse of sensitive persons, more especially observable when they are not in a state of agitation. Napoleon's pulse is said to have been but forty-five per minute; moreover, the heart's contractility was so little marked in him, that the action of this organ could scarcely be felt on applying the hand to the chest, even before the occurrence of that embonpoint into which he fell in later years.

It happens, however, that the circulation occasionally seems to increase its activity without any known cause. There is then what physicians call a *nervous pulse,* an appellation as just as it is true. This pulse characterises mere nervous excitement of the circulation. So true is this, that experienced practitioners are extremely cautious, in this case, in bleeding largely if the subject be weakly, experience having taught them that this increase of action was but apparent.

This singularity of the circulation, joined to diminished contractility of the heart and vessels, and consequent diminution of the velocity of the blood even from its very outset, produces another phenomenon which must not be overlooked, and that is, the unequal distribution of this fluid. The head, abdomen, and principal viscera, are sometimes in a state of plethora, whilst the blood cannot reach the surface and extremities without difficulty. Projected with but little force by the heart, circulating slowly, either from want of energy in the vis a tergo, or defect of tonicity in the capillary vessels, the blood seldom tarries on the exterior of the body. Thence, independently of other causes, the frequency of visceral congestions, coldness of the extremities, so distressing to studious and sedentary persons; thence, also, that habitual paleness so general indeed, that one of the fathers of the church calls it the beauteous complexion of great men.—*Pulchrum sublimium virorum florem.* (S. Greg. Naz. orat. 14.)

The respiration equally participates in the effects of this organic state. If it be true that the entire mass of blood passes about twelve times an hour through the heart and lungs, the expansion of the thorax must be performed with promptitude, facility, and in suitable proportions. But the weak state of the muscles, the frequent spasmodic constriction of the chest, diminish the extent of this cavity. These constrictions sometimes produce so sudden a reflux of blood into the heart and lungs, as to cause instant death. Thus Moliere died of pulmonary apoplexy. The effects, however, are generally more slow in developing themselves. The blood, impeded in its course, stagnates in the pulmonary parenchyma, it presses on, and gradually breaks down the meshes of this tissue; it separates and severs the fibres of the heart. Such is the origin of an infinite number of diseases, such as hæmoptysis, latent inflammations, aneurysms, &c. The oxygenation of this fluid is also imperfect, and venous plethora manifests itself at an early period with all its annoyances. Thus then it will, nay it must, be admitted, that the capacity of the thorax, the extent of respiration, a free and easy pulmonary circulation, regularity in the movements of the heart and arterial trunks, the reparation of the blood by the oxygen of the atmosphere, depend in a great measure on the development of muscular power.

Even the secretions and the functions of absorption are under the influence of contractility, as most of them become languid, when the energy of this property is diminished. With respect to animal heat and its various modifications, though the same causes cannot be assigned, still it must be evident that the calorific phenomena of the animal economy are closely and intimately connected with nervous action. Certain it is that, when there is an extreme predominance of this action, the animal heat has what may be called a special character: it is acrid, biting, and irregular; it is that *nervous heat,* remarkable more especially at a certain age, and widely different from that moist, mild, uniform heat, which generally attends the sanguineous temperament, and the period of youth.

On now passing to the external appearance of the body, we shall find the clearest signs of the exaggerated influence of sensibility over contractility. Every one feels anxious to see the person of a distinguished man—and yet how seldom does the reality correspond with the idea previously formed of him. The great man, on a close inspection, is seldom the man of his works; we look for him, if one may say so, when he is present: *adeo ut plerique, viso eo, quærant famam, pauci interpretentur.* In such cases it is obvious that the material element having been consumed, the body presents but a mere shadow of an appearance, exhausted as it is by the violence of the sensations and activity of the mind. Most great men, with few exceptions, are small in stature and in appearance, more especially when they have attained a certain age. Their feeble arms announce that we must seek elsewhere for the cause of their power: in a word, all their exterior bears the imprint of a feeble organization, which has sadly changed, and which has suffered deeply. There sometimes may be observed, in such cases, an invincible repugnance to exertion; whilst at other times the opposite feelings are evinced, but only by starts and temporarily. Sometimes the skin is devoid of colour and pale, the muscular fibre soft and flabby; whilst at other times the integuments are of a dark yellow colour, and the muscular fibre dry and somewhat tense; but it is rare to find, even in northern countries, that brightness of the physiognomy, that rosy tint, that character of freshness and of life, which indicate blooming health, and a full and free circulation. The body is often emaciated; yet sometimes we may observe extreme embonpoint at a very early period, a sure symptom of premature debility, as in the case of Gibbon, Fred. Schlegel, and Napoleon. Voltaire exhibited in his own person a striking ensemble of all the effects of an overwrought sensibility. "His emaciated form," as M. de Segur says of him, "retraced to my mind his laborious works......... his piercing eye sparkled with genius and archness; one might see in them at one and the same time the tragic poet, the author of *Œdipus* and of *Mahomet*, the profound philosopher, the ingenious romancist, that mind which so closely observed and so well satirized the human race. His *attenuated* and *curved* body was but a thin, almost transparent covering, through which his very soul and genius seemed to appear."

Yet, as our author well observes, it is not always that the exterior of illustrious men indicates what they really are; one cannot always divine that mobile, that impassioned temperament under the fleshy covering which encompasses them. In no case is that well-known expression of *volcano covered with snow* more applicable than here. Bonaparte, who so long presided over the destinies of France and Europe, exhibited nothing in his youth which could afford any indication of what he was to be. The same may be said of other illustrious men.

The physiognomy alone is often sufficient to discover the man, to whom Nature has been prodigal of her gifts. A broad, angular forehead, furrowed by the traces of grand and sublime thoughts, and eyes sparkling with fire, communicate to the countenance an animated expression, and surely with such a countenance, "marked," as Lavater says, "with the finger of God," the man cannot be a fool. And yet there are numerous exceptions to this law. Men of vast minds have been often observed to have but a very inexpressive physiognomy, as is evident in the case of Cromwell, Churchill, Johnson, and more particularly Goldsmith.

We must not, therefore, suppose that the outward appearance of distinguished men corresponds exactly with their genius and intellectual endowments, though such a thing often happens. The long sustained activity of the sensitive powers, the continued course of reflection and meditation, ordinarily concentrate the vital powers in the brain and internal functions, whereby the organs of motion, the source of physical power, gradually lose their size and energy; the body becomes debilitated, and no longer corresponds with the internal activity of the mind.

Such are the effects produced on the organization by the preponderance of the sensitive powers over the powers of motion.

Our author next proceeds to consider the effects of this law already stated on the intelligence in general, and commences with a remark of Pascal's, already referred to, pertinent to his subject; namely, that "we must not mistake ourselves; we are body as well as spirit." The study of man, he says, considered in his phenomena of organization, proves that the moral sensibility is, as one might say, the consequence and reflection of organic sensibility. This principle cannot be invalidated ; it is fully borne out and supported by anatomical research, by the laws of organization, by pathological phenomena, and by the lives of distinguished men. Thus, then, a very active and well-developed nervous system imparts to the mind a great power of manifestation. Every man in whom this apparatus, as well as the principal centres which compose it, predominate, presents to the observer an order of phenomena as extended as they are varied, in their succession as also in their modifications.

Without dwelling on particulars, we may first remark that sensibility, taken in its fullest development, presents—

1st. The capability of feeling;
2nd. The capability of knowing;
3rd. The capability of expression.

These three forms of one and the same power present striking differences in their action. The first is simply passive; there is merely a transmission of impressions; the second requires a certain activity of the brain; whilst the third, pre-eminently active, is the complement and acmé of intelligence; whence it is that it is much more rarely found in a high degree of perfection, than either of the other two. Most men feel, and even keenly, but the capability of giving expression to those feelings is given only to a very small number, to the few, *quos æquus amavit Jupiter*. Some philosopher has said, " if we were obliged, when writing, fully to satisfy ourselves, I do not think we would write one page in our whole life. We admire the Æneid, and justly, and yet Virgil wished to burn it." Voltaire said that he should die without having written one piece to his own taste or satisfaction. Those who are most gifted in this respect, never express all what they feel and as they feel. Thus unfortunately genius is always cramped from the insufficiency of the means of expression. So that the same organic constitution, which renders the individual susceptible of strong emotions, is not always sufficient to enable him to present the image of them externally. The man of genius lives and dies, tormented with the inability to reproduce the type of ideal perfection which he feels. Eternally confined and restricted by material possibilities, he is worried and worn out in his fruitless efforts to attain them. He desires to bathe in the waves of celestial light, but he sees these waves for ever retiring from him. So that in the arts of the imagination the most difficult point is not to think or to invent, but to embody those thoughts, so as that they may strike and captivate. Still organic sensibility is the prime basis of genius and talent, as it is there we must seek for the first element of sentiment and inspiration. From this sensibility that electric spark is given off, which arouses and inflames the ideas, thoughts, and passions. " Those germs of eloquence which reside and ferment in the bottom of the soul, and afterwards dart forth in such rapid flashes; that powerful, enrapturing eloquence, which electrifies from heart to heart the inert crowd, and raises it, as Jupiter raised the gods, those thoughts of fire, those animated words, which heat the imagination and subdue the understanding, are, after all, but the impetuous and ardent movements of a privileged and strongly stimulated sensibility."

Thus the action of the nervous apparatus is constantly represented to our eyes. This action is so marked in the great moral perturbations, that it influences the play of the other functions. Thus the conditions of the system being no longer in due proportion, all the springs and departments of life participate in this activity of the nervous system. It is remarkable also, that the metaphorical language of all nations represents with exactness and precision the effects produced on the œconomy by an exalted sensibility. "The blood freezes, the eyes sparkle, the heart burns, we shake and tremble with fear or hope; we are pale with terror, swollen with pride, panting with desire, &c." In a word, the organic disturbances and the intellectual emotions are perfectly proportioned, evidently because their source or origin is identical.

The second part of the work is devoted to the subject of what may be called pathological physiology, or the phenomena of life in the state of disease, and commences with the—

*Predisposing or General Causes of Diseases of Persons devoted to intellectual Labour.*—The first of these causes set down by our author is a delicate, mobile, impressible organisation, where the feeling of life is almost always exalted, where the sympathies are rapid, active, and multiplied; a cerebral system constantly kept in a state of permanent erethism by extra-normal and disturbing excitations. The next predisposing cause is the deficiency, or, at least, the progressive diminution of contractility; so that the individual loses the power of re-action, that is, the faculty of effectually repelling or neutralising the injurious action of several modifying agents. This, he says, is the fundamental character of this constitution, as has been already shown.

The third predisposing cause of disease in persons of this constitution is the unequal distribution of the vital and sensitive forces. If in such individuals some of the organs are in a state of perpetual action, other organs are condemned to a state of almost absolute inactivity. The hurried and abnormal action of innervation further presents a character of irregularity which is opposed to the equilibrium of the vital acts. The portion of nervous power which appertains to the functions of digestion, nutrition, circulation, &c. is for the most part directed to the mind, and to its organ, the brain. Some organs have a superfluous supply, while others do not receive enough; whence the organic relations necessary for the maintenance of health are actually destroyed. We next come to consider the—

*External or Secondary Causes.*—And the first stated is *sedentary life*—the second, *the want of pure air*—third, *prolonged and repeated watchings*—fourth, *certain positions* or *postures* of the body when at study—fifth, *the retention of urine and of the fæces*—sixth, *errors in diet*—seventh, *solitude*—eighth, *eccentric habits.* The influence of the first of these causes in the production of bad health is universally acknowledged—nor will that of the second, viz. the want of fresh air frequently renewed, be denied by many. The author here ridicules the poet who attempts to sing the beauties of Nature and the delights of rural life, while he at the same time inhabits some dirty narrow street or court from whence he scarcely ever emerges—and the painter, who attempts to paint *Aurora opening with roseate fingers the portals of the East,* though he scarcely ever sees the sun rise for whole months together. The mischievous effects of prolonged and repeated watching will scarcely be questioned. It, on the one hand, deprives the body of rest, over-excites the brain's action, augments the enormous wasting of the nervous principle, occasions sanguineous congestion in the head; and, on the other hand, it prevents the due reparation of the vital powers, or at least causes such reparation to be imperfect. The brain is so excited on such occasions, that oftentimes, when the exhausted thinker gives up his study and retires to rest, sleep flies his eyelids; the excitement still continues, and repose comes not. The state of cerebral tension, so desirable for the production and combination of ideas, still continues, notwithstanding all his efforts to diminish it; and, should sleep come at length, it is restless and disturbed, and recruits but imperfectly the exhausted strength. With respect to the position usually assumed by sedentary persons when at study, we may remark that, besides the curvature of the spine so often observable in such persons, such position interferes very much with the circulation of the blood, favours abdominal congestions, compresses the liver and stomach, and injures the functions of these organs. When the head is deeply involved and occupied in meditation, the other organs in vain appeal to the brain, to apprise it of their condition and warn it of their wants; the sensation is either wholly absent, or very slight. The brain is at work, the ideas flow on, the pen runs rapidly over the paper, and the individual defers to another time the business of eating, drinking, emptying the bowels, &c. What is the result of this? Debility of stomach, pulmonary congestions, catarrh and calculi of the bladder, obstinate constipation, &c.; in a word, a crowd of diseases depending on the individual constitution. The errors and deviations in diet to which literary men are occasionally liable; the alternate privations and excesses endured and indulged in by them, are well known. On this we shall only remark that, what is moderation in an ordinary man of the world, becomes excess in the man of study, whose extreme organic sensibility requires the strictest care. With respect to the next cause, scil. *solitude*, it is well known what charms this has for studious persons, charms to which people of the world are downright strangers. There is, however, considerable caution necessary to guard against the effect of this indulgence. The perpetual convergence of the vital movements towards the head, the uninterrupted activity of the brain, that force of thought, and series of ideas, reasonings, and inductions, which keep the brain in, as it were, a constant state of erection, distress, and harass beyond measure the springs of the œconomy. To sacrifice the flesh to the spirit may no doubt be good for fame, but it is always ruin to health. External impressions or distinctions have, on the contrary, the effect of preventing the mischievous effects of too long-continued solitude: they interrupt those disastrous concentrations of mind, distribute the vital forces equally, and impart to the circulation a uniform motion.

With respect to *eccentric habits* as a cause of disease, he cites several instances of literary men, who paid the forfeit of health and life for their indulgence in them. One of the instances, Bourdelin, an eminent physician, was so carried away by the allurements of study, that, wishing to devote a portion of the night to it, he gorged himself with coffee during the day, and then took opium, when he wished to have some sleep. The consequence was, a premature death. Among the other eccentricities of literary men he mentions the inordinate use of snuff and tobacco. Other individuals have been known to plunge their feet into cold water for the purpose of determining a rush of blood to the head, and of thereby exciting the powers of the mind. It is well known that certain faculties of the mind, as memory, are much increased during delirium, the paroxysms of fever, and other affections which determine more blood than usual to the head. Intoxication has been found to increase the energy of the intellectual faculties, and to revive the memory. We believe it is Mr. Combe who mentions the case of a porter, who, in a state of drunkenness, left the parcel he was employed to carry at the wrong house, and when sober, could not recollect what he had done with it. The next time, however, he became drunk, he recollected perfectly where he had left it. The author of the "Confessions of an English Opium Eater," observes, "that wine, up to a certain point, rather tends to steady the intellect, and that a few glasses rather advantageously affected his own." This, of course, shows that whatever cause produces an increased afflux of blood to the head has the effect also of heightening the intellectual powers: it shows further, and more generally, that the varying states of the organization have a powerful influence on the intellectual and moral faculties. We must, however, remember that, to affect the mind beneficially, to increase its energy and render such energy permanent, it is indispensably necessary to give constant attention to the agents that act on the body, and to take care that they injure not the mind by too much excitement of the physical system, nor prevent the proper development of its powers by too little; for wine and all other stimuli, though they may for a while give increased energy to the intellect, ultimately depress and weaken it. In concluding this portion of our analysis we shall only remark, what indeed we have already said, that these secondary causes of disease in deep thinkers have but a relative influence, their intensity being directly proportioned to the physical powers of the individual on whom they act.

Our athor next proceeds to consider the *organs more especially affected by excessive intellectual exertion.* If, he says, there be a positive fact in pathology, it is, that all the causes capable of producing irritation and inflammation, commence by exciting and increasing sensibility. This pathological principle we remember to have seen strikingly and most satisfactorily illustrated in Dr. Billing's work.* The propagation of nervous irritation is extremely remarkable in the sensitive constitution now under consideration. It is then on the nervous system, generally and primarily, that all the causes of disease act. Now, when this system has acquired an exclusive and unnatural predominance, when the economy is, as one may say, saturated with irritability, it is clear that all the organs which it pervades must be in a morbid state, that is, must be extremely disposed to pathological affections. This is precisely what takes place in several artists, men of letters, statesmen, &c. devoted to the engrossing and tyrannical occupations of the mind. There are, however, certain organs more especially disposed to the action of those causes, and to which the reader's attention is now called. And first,

* First Principles of Medicine, by A. Billing, M.D.

*The Brain.*—The indisputable supremacy of this organ is the same under all the modifications which the economy may undergo; it is in fact always the first power of organic association. But here this superiority, and the dangers consequent on it, are increased by the excessive activity to which the organ is subjected. It is certainly in the brain and in its acts, that we must seek the source of happiness, the instrument of the indescribable pleasures and inconceivable delights of those men who live solely by thought; unfortunately too, it is here we must seek the true *atrium mortis*, the origin of those evils to which they are exposed. If we but take into consideration the high importance of the functions of the brain, the extent of its relations, the energy and diversity of its sympathetic connexions, we shall no longer feel surprised at the number, the variety, or severity of those diseases which are occasioned by its extreme and incessant excitation. The integrity of its functions forms the basis of health; without such integrity all is wrong. It may here be observed, that there exist many, very many shades in the changes produced in the encephalon, shades oftentimes inappreciable; for we judge and recognize only the extreme cases. It may be readily conceived that incessant meditation and exertion of the mind, which stretch and strain the springs of thought, absorb life and devour it by little and little, constantly keeping the cerebral powers in a state of super-excitement, must ultimately determine an inflammatory *molimen*, or else a state of general debility, which is sure to produce serious changes in the organ.

Now these changes are sometimes slow, sometimes rapid. Latent irritations, slow chronic inflammations, partial congestions, softening of several points of the cerebral substance, frequently develop themselves only by equivocal symptoms of morbid excitement; as the evil advances, the accompanying phenomena clearly manifest the cause, but the time for remedying this state is now gone by. The circumstances of temperament, age, &c. obviously influence the cerebral changes which occur. Young persons are more liable to inflammations of the membranes; aged persons, in whom the venous plethora predominates, frequently suffer organic lesions, congestions, ruptures of the vessels of this viscus, ramollissement, &c. In all, however, the pathological affections of the brain are always of a particularly serious character, by reason of the intense and incessant excitements which this organ undergoes. The moral sensibility, also, as well as the physical apparatus, acquires an extraordinary increase of activity. If it be true that, in civilized men, the imagination centuples the causes and results of diseases, what must be the effect which this imagination produces in men who concentrate their existence in the exercise of the intellectual faculties! One of the principal effects of the continued tension of the brain is to weaken all the organs more or less dependent on it, by depriving them of a part of the nervous influx necessary to their action. How true that saying of the distinguished physician of Catherine de Medicis, Fernel:—*A capite fluit omne malum.*

The organ most exposed, perhaps, to this privation is the *stomach:* debility of the digestive system seems in a manner peculiar to illustrious men. Some have even gone to the ridiculous extent of estimating a man's genius by the state of his stomach. We must, however, acknowledge the truth or Tissot's assertion, that "the man who thinks most, digests worst, cæteris paribus, and that he who thinks least is the man who digests best." Daily experience, and the history of distinguished men, afford abundant proofs of the truth of this assertion. Some, however, attribute this delicacy of stomach in profound thinkers to their sedentary life; that this may in part account for the phenomenon we admit; that it will not account for the matter perfectly, is evident from the good digestive powers possessed by women, artisans, and others who also lead sedentary lives.

Napoleon, whose astonishing activity of mind surprised his cotemporaries, had, on the contrary, a very susceptible and irritable stomach. Our author has already shewn, that when sensibility predominates, contractility diminishes; and this occurs more especially with respect to the digestive apparatus, the tonic and contractile power of which is not always proportioned to its sensibility; the consequence of this is, that the debility of the stomach now in question is always accompanied with nervous irritation of this organ. To this we may add, that the continued excitement of the brain has a direct and immediate influence on the stomach. It is well known that a strong exertion of mind, sudden news, agreeable or otherwise, at once disturbs digestion, suspends the appetite, and throws the digestive organs into an almost morbid state of languor, and as every thing is linked and connected in the system, such a state, being continued for a length of time, re-acts on the other organs. When the act of digestion is interfered with and retarded; when chylification is tedious, or incomplete, it is evident that such imperfect elaboration of the chyle will introduce into the system nothing but impoverished blood, and that its nutrition will be essentially altered. The body then becomes weaker and weaker; the flesh becomes devoid of life; the tissues without consistence, whilst the sensibility increases in proportion, and the nervous irritability becomes more developed. Improve, however, the digestion, let the nutrition be made more perfect, and then the blood becomes pure and rich, the body soon becomes strengthened, the sensibility, both physical and moral, remains within due limits. Thus we see the sphere of the stomach's activity is very extended, independently of its connexions with the nervous plexuses surrounding it; whence it is that the epigastrium is one of the principal points of concentration of the vital influences. In this sense it was we are to suppose that Wepfer called this viscus *præses systematis nervosi.* Amatus, a Portuguese physician, it was, who said that a bad stomach follows profound thinkers as faithfully as the shadow follows the body.

Next to the stomach, our author sets down the *liver* as one of the organs most frequently modified in its functions, and even in its structure, in men of studious, and sedentary habits. The predominating venous plethora existing in the abdomen, the complicated structure of this viscus, as ascertained by the recent valuable researches of Kiernan, researches by the way not tending to gratify mere anatomical curiosity, but calculated to afford sound practical indications in treating the diseases of this viscus, the close sympathies subsisting between this viscus and the stomach through the ganglionic system, its connexion also with the brain, all these considerations at once account for the frequency of lesions of this organ, its engorgements and tumefactions, its inflammations, sometimes of a slow, chronic character, and sometimes rapid and fatal. History tells us that the poet Racine died of an abscess of the liver which was neglected, or mistaken for some other affection. The importance of a healthy secretion of bile, depending as it does on a healthy state of this organ, is obvious. Another effect of hepatic disturbance or disease, by interfering with the abdominal circulation, is hæmorrhoids, that scourge of studious and sedentary persons.

Next to the liver the *urinary organs* appear to be the part of the system most affected in literary persons—and next, the senses of *hearing* and of *sight.*

Our author, after enumerating the vital organs generally affected in studious men, next proceeds to enumerate the *diseases,* at least the principal diseases, to which these persons are liable. In this enumeration he follows the order of the different organs, and accordingly begins with *affections of the brain.* These, he remarks, sometimes come on rapidly, and explode, as in cerebral inflammations and brain fevers; whilst the effects of incessant mental toil are at other times slow. Apoplexy itself, to which so many profound thinkers fall victims, presents various modifications. Before the person gets the fatal stroke, how often has the brain been excited, strained, and outraged! how many times have *rushes* of blood to the head, squalls of heat in the face, dull pains and sense of weight in the frontal region, temporary dimness, violent arterial pulsations in the temporal region, and restless sleep, clearly indicated sanguineous repletion, and cerebral excitement beyond what was natural. These effects, however, pass away; they are forgotten; they return, and the delicate structure of the brain is soon broken up. The author here enumerates a list of distinguished men, great intellectual labourers, who fell victims to apoplexy; or some other disease of the brain. Among the rest he mentions Swift, Petrarch, Copernicus, Malpighi, Richardson, Spallanzani, Monge, Cabanis, Corvisart, and Sir Walter Scott. A slight attack of this affection has been called by Ménage *un brevet de retenue de mort;* that is, as we may render it, *Death's bond of security.* Napoleon, who dreaded apoplexy, one day asked Corvisart, his first physician, for some information respecting this disease. "Sire," replied Corvisart, "apoplexy is always dangerous, but it is preceded by certain symptoms. Nature seldom strikes the blow without giving warning. A first attack, which is almost always slight, is a *summons without costs* (sommation sans frais;) a second a *summons with costs* (—— avec frais;) but a third, is an *execution on the person* (prise de corps.)" Corvisart himself afforded a melancholy proof of the truth of his assertion.

Our author thus endeavours to explain the gradual action of the causes of this disease. The permanent excitements of the brain at first increase its energy, or activity, in fact, its *vitality.* This excess of action, when repeated, occasions every time an afflux of blood to this organ; the stimulations then become congestional. At first these congestions disappear more or less completely, the brain is freed, and the equilibrium is restored. Afterwards the forced dilatations of the vessels become such that the congestions disappear but imperfectly; this gives rise to symptoms not, however, of a very very alarming nature. At a still later period, when age advances, and the venous system increases in size, and the cerebral veins have a tendency to become varicose, at the same time that the arteries diminish in diameter, these congestions become permanent. This state of engorgement increases rapidly, if there be aneurysm of the heart. From these morbid states arise coma, stupor, softening of the brain, tremors, paralysis, and, finally, apoplexy in all its degrees.

It sometimes happens, after prolonged study and watching, that the action of the brain becomes totally suspended. The painful torpor of the nervous apparatus which is the result of this, renders the individual incapable of connecting two ideas, in fact, thought ceases altogether.

Now, while the vital action is thus extreme in the encephalon, the digestive apparatus becomes altogether languid. The abdominal circulation which is naturally not very active, especially in the branches of the vena portæ, so happily called by some one *porta malorum*, now becomes very much impeded. The afflux of arterial blood towards the upper parts of the body; the sedentary life, the habitual flexion of the trunk, so frequent among studious persons, contribute still more to increase the evil. During this time the stomach loses its contractile power, a distressing sensibility becomes developed in this viscus, and the digestive function becomes more and more impaired. When one becomes excessively attentive to his digestive powers, when the stomach is delicate, scrupulous, and requires some particular kind of food, when the appetite is irregular, when flatulence exists with sour eructations, a feeling of burning heat in the throat, swelling and heat in the epigastrium during the act of digestion, our precautions must be redoubled. It is certain that then the alimentary tube is threatening some serious disease, which sooner or later will explode. Inflammation of the liver and stomach, in all its stages, jaundice, gastralgia, spontaneous perforations, nervous colics, frequent vomiting, scirrhous pylorus, cancerous affections, &c. are the results of the morbific principle now under consideration. And, making the same observation here as when speaking of the brain, we find that slight diseases of the digestive apparatus, such as loss of appetite, painful digestion, flatulence, are shades of organic and functional changes which often lead to lesions no longer to be remedied by art. Though *constipation* is not, strictly speaking, a disease, it occurs so frequently in the class of persons we are now considering, it is the latent or obvious cause of so many diseases, that itself may well be considered one. Two causes produce it, heat and unnatural dryness of the intestinal canal, or else debility and atony of this same viscus. The latter cause is remarkable enough in aged persons. It would be superfluous to enumerate all the consequences produced by obstinate constipation. We shall mention merely the principal of them; these are, inflammation of the intestinal canal, degeneration of tissue, hæmorrhoids, fistula in ano, &c.

*Calculi in the kidneys and bladder*.—This is a frequent attendant on sedentary and studious persons—Erasmus, Luther, Bossuet, Buffon, were victims to this visitation. *Hypochondriasis—melancholy—monomania*, cum multis aliis, may be added to the list.

We have now presented our readers with an analysis of the physiological and pathological parts of this certainly extraordinary work. Many of our author's views are novel; many of them are interesting, and perhaps truth will oblige us to say, some of them border very closely on what our German friends would call the transcendental philosophy. In our sketch we have endeavoured to present the author's opinions in as tangible and matter-of-fact a form as we could, though we fear much we have not been as successful in our efforts to materialise and give a body to the abstractions of his mind, as he has been in giving a soul to what ordinary men would consider the objects of mere sense. The English reader, however, accustomed as he is to plain, common-sense facts and reasonings, when he finds any thing here not very intelligible, must be content to take *omne ignotum pro magnifico,* or must do as the school-master mentioned by Quintilian, who, whenever he found any thing unintelligible in the literary compositions of his scholars, encouragingly exclaimed, *tanto melius, ipse non intelligo!* With the therapeutical part of the work we must decline meddling; all that is good in it we have ourselves, and with the rest we can very well dispense.

